# Improving CT Image Tumor Segmentation Through Deep Supervision and Attentional Gates

**DOI:** 10.3389/frobt.2020.00106

**Published:** 2020-08-28

**Authors:** Alžběta Turečková, Tomáš Tureček, Zuzana Komínková Oplatková, Antonio Rodríguez-Sánchez

**Affiliations:** ^1^Artificial Intelligence Laboratory, Faculty of Applied Informatics, Tomas Bata University in Zlin, Zlin, Czechia; ^2^Intelligent and Interactive Systems, Department of Computer Science, University of Innsbruck, Innsbruck, Austria

**Keywords:** medical image segmentation, CNN, UNet, VNet, attention gates, deep supervision, tumor segmentation, organ segmentation

## Abstract

Computer Tomography (CT) is an imaging procedure that combines many X-ray measurements taken from different angles. The segmentation of areas in the CT images provides a valuable aid to physicians and radiologists in order to better provide a patient diagnose. The CT scans of a body torso usually include different neighboring internal body organs. Deep learning has become the state-of-the-art in medical image segmentation. For such techniques, in order to perform a successful segmentation, it is of great importance that the network learns to focus on the organ of interest and surrounding structures and also that the network can detect target regions of different sizes. In this paper, we propose the extension of a popular deep learning methodology, Convolutional Neural Networks (CNN), by including deep supervision and attention gates. Our experimental evaluation shows that the inclusion of attention and deep supervision results in consistent improvement of the tumor prediction accuracy across the different datasets and training sizes while adding minimal computational overhead.

## 1. Introduction

The daily work of a radiologist consists of visually analyzing multiple anatomical structures in medical images. Subtle variations in size, shape, or structure may be a sign of disease and can help to confirm or discard a particular diagnosis. However, manual measurements are time-consuming and could result in inter-operator and intra-operator variability (Sharma and Aggarwal, 2010; Jimenez-del-Toro et al., 2016). At the same time, the amount of data acquired via Computer tomography (CT) and Magnetic resonance (MR) is ever-growing (Sharma and Aggarwal, 2010). As a result, there is an increasing interest in reliable automatic systems that assist radiological experts in clinical diagnosis and treatment planning. One of such aids to experts is medical image segmentation, which consists of voxel-wise annotation of target structures in the image and it is present in many recent research work. Yearly medical image competition challenges^1^ allow to the computer vision and machine learning experts to access and evaluate medical image data (Jimenez-del-Toro et al., 2016).

Deep learning techniques, especially convolutional neural networks (CNN), have become the state-of-the-art for medical image segmentation. Fully convolutional networks (FCNs) (Long et al., 2015) and the U-Net (Ronneberger et al., 2015) are two of the most commonly used architectures. Their area of application includes anatomical segmentation of cardiac CT (Zreik et al., 2016), detection of lung nodules in chest CT (Hamidian et al., 2017), multi-organ segmentation in CT and MRI images of the abdomen (Jimenez-del-Toro et al., 2016), and ischemic stroke lesion outcome prediction based on multispectral MRI (Winzeck et al., 2018) among others.

Despite the success of deep CNN techniques, there are difficulties inherent to their applicability. First, large datasets are needed for the successful training of deep CNN models. In medical imaging, this may be problematic due to the cost of acquisition, data anonymization techniques, etc. Second, volumetric medical image data require vast computational resources, even when using graphical computation units (GPU) the training process is very time-consuming. Therefore, every new proposal should take into account not only the performance but also the computational load.

Current CT-based clinical abdominal diagnosis relies on the comprehensive analysis of groups of organs, and the quantitative measures of volumes, shapes, and others, which are usually indicators of disorders. Computer-aided diagnosis and medical image analysis traditionally focus on organ or disease based applications, i.e., multi-organ segmentation from abdominal CT (Jimenez-del-Toro et al., 2016; Hu et al., 2017; Gibson et al., 2018), or tumor segmentation in the liver (Linguraru et al., 2012), the pancreas (Isensee et al., 2018), or the kidney (Yang et al., 2018).

There are two significant challenges in automatic abdominal organ segmentation from CT images (Hu et al., 2017). One of such challenges is how to automatically locate the anatomical structures in the target image because different organs lay close to each other and can also overlap. Moreover, among individual patients exists considerable variations in the location, shape, and size of organs. Furthermore, abdominal organs are characteristically represented by similar intensity voxels as identify surrounding tissues in CT images. The other challenge is to determine the fuzzy boundaries between neighboring organs and soft tissues surrounding them.

The task of detecting cancerous tissue in an abdominal organ is even more difficult because of the large variability of tumors in size, position, and morphology structure. Results are quite impressive when the focus is on detecting organs; an example of this is (Isensee et al., 2018), achieving dice scores of 95.43 and 79.30 for liver and pancreas segmentation. On the other hand, these values drop dramatically when the focus is on detecting the tumor, where values are as low as 61.82 and 52.12 for their respective (liver and pancreas) tumor classes. There is also a high variability on tumor classification depending on the organ, e.g., Yang et al. (2018) presents dice scores of 93.1 and 80.2 when the organ is the kidney and its tumor detection, respectively.

On the other hand, all the organs have a typical shape, structure, and relative position in the abdomen. The model could then benefit from an attentional mechanism consolidated in the network architecture, which could help to focus specifically on the organ of interest. For this purpose, we incorporated the idea of attention gates (AG) (Oktay et al., 2018). Attention gates identify salient image regions and prune feature responses to preserve only the activations relevant to the specific task and to suppress feature responses in irrelevant background regions without the requirement to crop the region of interest.

Many research papers have incorporated attention into artificial CNN visual models for image captioning (Xu et al., 2015), classification (Mnih et al., 2014; Xiao et al., 2015), and segmentation (Chen et al., 2016). In the case of Recurrent Neural Networks (RNN), Ypsilantis and Montana (2017) presents an RNN model that learns to sequentially sample the entire X-ray image and focus only on salient areas. In these models, attention could be divided into two categories: hard and soft attention. As described by Xu et al. (2015), hard attention is when the attention scores are used to select a single hidden state, e.g., iterative region proposal and cropping. Such an attention mechanism is often non-differentiable and relies on reinforcement learning for updating parameter values, which makes training quite challenging. On the other hand, soft attention calculates the context vector as a weighted sum of the encoder hidden states (feature vectors). Thus, soft attention is differentiable, and the entire model is trainable by back-propagation. The attention modules which generate attention-aware features presented by Wang et al. (2017) was the state-of-the-art object recognition performance on ImageNet in 2017. Huang et al. (2019) presents a Criss-Cross Network (CCNet) with a criss-cross attention module and achieves the state-of-the-art results of mIoU score of 81.4 and 45.22 on Cityscapes test set and ADE20K validation set, respectively. Grewal et al. (2018) combines deep CNN architecture with the components of attention for slice level predictions and achieves 81.82% accuracy for the prediction of hemorrhage from 3D CT scans, matching the performance of a human radiologist. Other boosted convolutional neural network with attention and deep supervision (DAB-CNN) (Kearney et al., 2019) achieves state-of-the-art results in automatic segmentation of the prostate, rectum, and penile bulb.

Deep supervision was firstly introduced by Lee et al. (2015) as a way to deal with the problem of the vanishing gradient in training deeper CNN for image classification. This method adds companion objective functions at each hidden layer in addition to the overall objective function at the output layer. Such a model can learn robust features even in the early layers; moreover the deep supervision brings some insight on the effect that intermediate layers may have on the overall model performance. Since then, deep supervision was successfully applied in many vision models. In the case of medical applications, it has been employed to prostate segmentation (Zhu et al., 2017), to the liver (Dou et al., 2016), and pancreatic cyst (Zhou et al., 2017) segmentation in CT volumes, and to brain tumor segmentation from magnetic resonance imaging (Isensee et al., 2017).

In the present work, we propose a methodology for a more reliable organ and tumor segmentation from computed tomography scans. The contribution of this work is three-fold:

- A methodology that achieves the state-of-the-art performance on several segmentation tasks dealing with organ and tumor segmentation, of special interest is the increase obtained in the precision of tumor segmentation.
- A visualization of the feature maps from our CNN architecture to provide some insight into what is the focus of attention in the different parts of the model for better tumor detection.
- Third and not last, we provide a novel and extended comparison of CNN architectures for different organ-tumor segmentation from abdomen CT scans.

## 2. Methodology

We will provide the details of the proposed methodology in this section. Firstly, we will explain the preprocessing and normalization of the medical image data. Secondly, we will provide a detailed description of the model architecture, the attention gates, and the deep supervision layers. The loss function, the optimizer, and other specifics of interest are detailed in the following subsection, which also describes patch sampling and data augmentation techniques utilized in order to prevent overfitting. The last part shortly outlines inference and how the image patches are stitched back together. We provide a publicly available implementation of our methodology using PyTorch at: github.com/tureckova/Abdomen-CT-Image-Segmentation.

### 2.1. Data Preprocessing

CT scans might be captured by different scanners in different medical clinics with nonidentical acquisition protocols; therefore the data preprocessing step is crucial to normalize the data in a way that enables the convolutional network to learn suitable and meaningful features properly. We preprocess the CT scan images as follows (Isensee et al., 2018):

- All patients are resampled to the median voxel spacing of the dataset using the third-order spline interpolation for image data and the nearest neighbor interpolation for the segmentation mask.
- The dataset is normalized by clipping to the [0.5, 99.5] percentiles of the intensity values occurring within the segmentation masks.
- Z-score normalization is applied based on the mean and standard deviation of all intensity values occurring within the segmentation masks.

Because of memory restrictions, the model was trained on 3D image patches. All the models were trained on an 11GB GPU. A base configuration of the input patch size of 128 × 128 × 128 and a batch size of 2 was chosen to fit our hardware set up. Then the model automatically adapts these parameters, so they reflect the median image size of each dataset. We consider two different approaches:

**Full-resolution**—the original resolutions of images are used for the training, and relatively small 3D patches are chosen randomly during training. This way, the network has access to high-resolution details; on the other hand, it neglects context information.
**Low-resolution**—the patient image is downsampled by a factor of two until the median shape of the resampled data has less than four times the voxels that can be processed as an input patch. 3D patches are also chosen randomly during training. In this case, the model has more information about the context but lacks high-resolution details.

### 2.2. Model Architecture

Deep learning techniques, especially convolutional neural networks, occupy the main interest of research in the area of medical image segmentation nowadays and outperform most techniques. A very popular convolution neural network architecture used in medical imaging is the encoder-decoder structure with skip connections at each image resolution level. The basic principle was firstly presented by Ronneberger et al. (2015) for segmenting 2D biomedical images; this network was named U-Net. U-Net traditionally uses the max-pooling to downsample the image in the encoder part and upsampling in the decoder part of the structure. The work of Milletari et al. (2016) extended the model for volumetric medical image segmentation and replaced the max-pooling and upsampling by convolutions, creating a fully convolutional neural network named V-Net. The original U-Net architecture was quickly extended into 3D, and since then, the literature seems to be using names U-Net and V-Net interchangeably. In this work, all models work with volumetric data, and we decided to keep the original architectures naming and differences:

- **UNet**—the encoder-decoder structure with the skip connections using the max-pooling to downsample the image in the encoder part and upsampling in the decoder part of the structure.
- **VNet**—the fully convolutional encoder-decoder architecture with skip connections.

We follow encoder-decoder architecture choices applied to each dataset by Isensee et al. (2018). We use 30 feature maps in the highest layers (the number of feature maps doubles with each downsampling), and we downsample the image along each axis until the feature maps have size 8 or for a maximum of 5 times. The encoder part consists of context modules, and the decoder part is composed of localization modules. Each module contains a convolution layer, a dropout layer, an instance normalization layer, and a leakyReLU.

**Figure 1:**
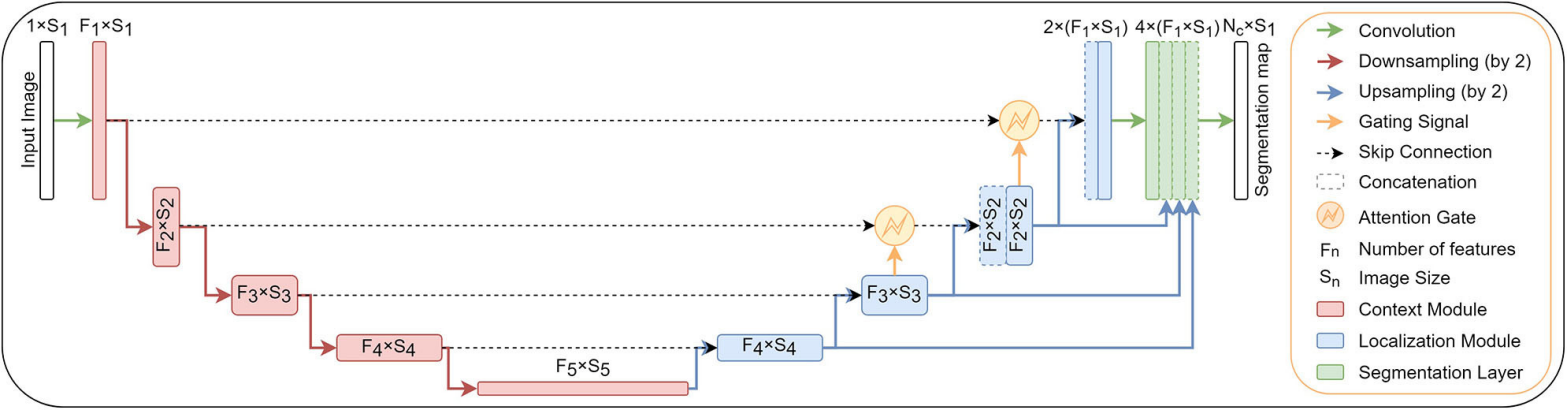
A block diagram of the segmentation model with attention gates and deep supervision.

In addition to original encoder-decoder network architecture, we add attention gates (Oktay et al., 2018) in the top two model levels and deep supervision (Kayalibay et al., 2017). Both extensions are described in the next two subsections. The structure of proposed network architecture is shown in Figure 1.

#### 2.2.1. Attention Gates

**Figure 2:**
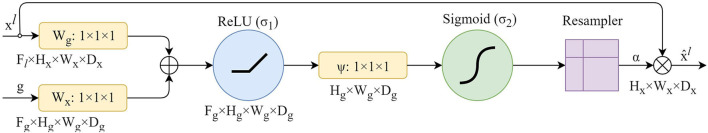
A block diagram of additive attention gate (AG) (Oktay et al., 2018). Input features (*x*^*l*^) are scaled with the attention coefficients (α) computed in AG. Spatial regions are selected by analyzing both the activations and the contextual information provided by the gating signal (*g*) which is collected from a coarser resolution scale. Attention coefficients are resampled to match the resolution of (*x*^*l*^) by trilinear interpolation.

Attention coefficients, α_*i*_ ∈ [0, 1] emphasizes salient image regions and significant features to preserve only relevant activations specific to the actual task. The output of AGs (1) is the element-wise multiplication of input feature-maps and attention coefficients:

(1) $$\begin{array}{l} {\hat{x}}_{i,c}^{l}=x_{i,c}^{l}\cdot \alpha_{i,c}^{l} \end{array}$$

where $\alpha_{i,c}^{l}$ is the attention coefficient (obtained using Equation 3, below), and $x_{i,c}^{l}$ is pixel *i* in layer *l* for class *c*. $x_{i}^{l}\in {\mathbb{R}}^{F_{l}}$ where *F*_*l*_ corresponds to the number of feature-maps in layer *l*. Therefore, each AG learns to focus on a subset of target structures. The structure of an attention gate is shown in Figure 2. A gating vector *g*_*i*_ is used for each pixel *i* to determine the regions of focus. The gating vector contains contextual information to reduce lower-level feature responses. The gate uses additive attention (2), formulated as follows (Oktay et al., 2018):

(2) $$\begin{array}{l} q_{att}^{l}=\psi^{T}\left(\sigma_{1}\left(W_{x}^{T}x_{i,c}^{l}+W_{g}^{T}g_{i,c}+b_{g}\right)\right)+b_{\psi } \end{array}$$

(3) $$\begin{array}{l} \alpha_{i,c}^{l}=\sigma_{2}\left(q_{att}^{l}\left(x_{i,c}^{l},g_{i,c},\Theta_{att}\right)\right), \end{array}$$

where $\sigma_{1}\left(x_{i,c}^{l}\right)=max\left(0,x_{i,c}^{l}\right)$ is rectified linear unit. AG is characterized by a set of parameters Θ_*att*_ containing: linear transformations $W_{x}\in {\mathbb{R}}^{F_{l}\times F_{int}}$, $W_{g}\in {\mathbb{R}}^{F_{g}\times F_{int}}$, $\psi \in {\mathbb{R}}^{F_{int}\times 1}$ and bias terms *b*_ψ_ ∈ ℝ, $b_{g}\in {\mathbb{R}}^{F_{int}}$. $\sigma_{2}\left(x_{i,c}^{l}\right)=\frac{1}{1+exp\left(-x_{i,c}^{l}\right)}$ corresponds to a sigmoid activation function. The linear transformations are computed using channel-wise 1 × 1 × 1 convolutions of the input tensors. All the AG parameters can be trained with the standard back-propagation updates.

#### 2.2.2. Deep Supervision

Deep supervision (Kayalibay et al., 2017) is the design where multiple segmentation maps are generated at different resolutions levels. The feature maps from each network level are transposed by 1 × 1 × 1 convolutions to create secondary segmentation maps. These are then combined in the following way: First, the segmentation map with the lowest resolution is upsampled with bilinear interpolation to have the same size as the second-lowest resolution segmentation map. The element-wise sum of the two maps is then upsampled and added to the third-lowes segmentation map and so on until we reach the highest resolution level. For illustration see Figure 1.

These additional segmentation maps do not primarily serve for any further refinement of the final segmentation map created at the last layer of the model because the context information is already provided by long skip connections. The secondary segmentation maps help in the speed of convergence by “encouraging” earlier layers of the network to produce better segmentation results. A similar principle has been used by Kayalibay et al. (2017) and Chen et al. (2018).

### 2.3. Training

Unless stated otherwise, all models are trained with a five-fold cross-validation. The network is trained with a combination of dice (5) and cross-entropy (6) loss function (4):

(4) $$\begin{array}{l} L_{total}=L_{dice}+L_{crossEntropy}, \end{array}$$

(5) $$\begin{array}{l} L_{dice}=-\frac{2}{\left|C\right|}\underset{c\in C}{\sum }\frac{\sum_{i\in I}u_{i}^{c}v_{i}^{k}}{\sum_{i\in I}u_{i}^{c}+\sum_{i\in I}v_{i}^{c}}, \end{array}$$

(6) $$\begin{array}{l} L_{crossEntropy}=-\underset{c\in C}{\sum }\underset{i\in I}{\sum }\left(v_{i}^{c}\text{log}\left(u_{i}^{k}\right)\right), \end{array}$$

where *u* is the softmax output of the network and *v* is a one hot encoding of the ground truth segmentation map^2^. Both *u* and *v* have shape *I* × *C* with *i* ∈ *I* being the number of pixels in the training patch/batch and *c* ∈ *C* being the classes. The cross-entropy loss speeds up the learning in the beginning of the training, while the dice loss function helps to deal with the label unbalance which is typical for medical images data.

The dice loss is computed for each class and each sample in the batch and averaged over the batch and over all classes. We use the Adam optimizer with an initial learning rate 3 × 10^−5^ and *l*_2_ weight decay 3 × 10^−5^ for all experiments. An epoch is defined as the iteration over all training images. Whenever the exponential moving average of the training loss does not improve within the last 30 epochs, the learning rate is decreased by a factor of 0.2. We train till the learning rate drops below 10^−6^ or 1, 000 epochs are exceeded.

Gradient updates are computed by standard backpropagation using a small batch size of 2. Initial weights values are extracted from a normal distribution (He et al., 2015). Gating parameters are initialized such that the attention gates let pass all feature vectors at all spatial locations.

#### 2.3.1. Data Augmentation and Patch Sampling

Training of the deep convolutional neural networks from limited training data suffers from overfitting. To minimize this problem, we apply a large variety of data augmentation techniques: random rotations, random scaling, random elastic deformations, gamma correction augmentation, and mirroring. All the augmentation techniques are applied on the fly during training. Data augmentation is realized with a framework which is publicly available at: https://github.com/MIC-DKFZ/batchgenerators.

The patches are generated randomly on the fly during the training, but we force that minimally one of the samples in a batch contains at least one foreground class to enhance the stability of the network training.

### 2.4. Inference

According to the training, inference of the final segmentation mask is also made patch-wise. The output accuracy is known to decrease toward the borders of the predicted image. Therefore, we overlap the patches by half the size of the patch and also weigh voxels close to the center higher than those close to the border, when aggregating predictions across patches. The weights are generated, so the center position in a patch is equal to one, and the boundary pixels are set to zero, in between the values are extracted from a Gaussian distribution with sigma equal to one-eight of patch size. To further increase the stability, we use test time data augmentation by mirroring all patches along all axes.

## 3. Experimental Evaluation and Discussion

In order to show the validity of the proposed segmentation method, we evaluate the methodology on challenging abdominal CT segmentation problem. We appraise the detection of cancerous tissue inside three different organs: pancreas, liver, and kidney.

### 3.1. CT Scan Datasets

The experiments are evaluated on three different CT abdominal datasets featuring organ and tumor segmentation classes: kidney, liver, and pancreas. Each dataset brings slightly different challenges for the model. More information about each task dataset, training setups, and concrete network topologies are as follows (see also Table 1).

**Table 1:** An overview of image shapes, training setups, and network topologies for each task.

		**High resolution**	**Low resolution**
Kidney	Num. images training	168	168
	Num. images validation	42	42
	Median patient shape	511 × 511 × 136	247 × 247 × 127
	Input patch size	160 × 160 × 48	128 × 128 × 80
	Num. downsampling per axis	5, 5, 3	5, 5, 4
	Batch size	2	2
Liver	Num. images training	105	105
	Num. images validation	26	26
	Median patient shape	482 × 512 × 512	189 × 201 × 201
	Input patch size	96 × 128 × 128	96 × 128 × 128
	Num. downsampling per axis	5, 5, 5	5, 5, 5
	Batch size	2	2
Pancreas	Num. images training	224	224
	Num. images validation	57	57
	Median patient shape	96 × 512 × 512	88 × 299 × 299
	Input patch size	40 × 192 × 160	64 × 128 × 128
	Num. downsampling per axis	3, 5, 5	3, 5, 5
	Batch size	2	2

#### 3.1.1. Kidney

The dataset features a collection of multi-phase CT imaging, segmentation masks, and comprehensive clinical outcomes for 300 patients who underwent nephrectomy for kidney tumors at the University of Minnesota Medical Center between 2010 and 2018 (Heller et al., 2019). Seventy percent (210) of these patients have been selected at random as the training set for the 2019 MICCAI KiTS Kidney Tumor Segmentation Challenge^3^ and have been released publicly.

We perform five-fold cross-validation during training: 42 images are used for validation and 168 images for training. The mean patient shape after the resampling is 511 × 511 × 136 pixels in case of high-resolution and 247 × 247 × 127 pixels in low-resolution. According to the median shapes, we use 5, 5, and 3 downsampling for each respective image axis in high-resolution and 5, 5, 4 downsamplings in low-resolution. The patch size in case of high-resolution is 160×160×48 pixels and 128×128× 80 pixels for low-resolution.

#### 3.1.2. Liver

The dataset features a collection of 201 portal-venous-phase CT scans and segmentation masks for liver and tumor captured at IRCAD Hôpitaux Universitaires. Sixty-five percent (131) of these images have been released publicly as the training set for the 2018 MICCAI Medical Decathlon Challenge^4^ (Simpson et al., 2019). This dataset contains a big label unbalance between organ (liver) and tumor. The inclusion of the dice term in the loss function (section 2.3) helps to mitigate the negative effects of such unbalance.

We perform five-fold cross-validation during training: 26 images are used for validation and 105 images for training. The mean patient shape after the resampling is 482 × 512 × 512 pixels in case of high-resolution and 189 × 201 × 201 pixels in low-resolution. According to the median shapes, we downsample five times each respective image axis in both high-resolution and low-resolution. The patch size in case of high-resolution was 96×128×128 pixels and 96×128×128 pixels for low-resolution.

#### 3.1.3. Pancreas

The dataset features a collection of 421 portal-venous-phase CT imaging and segmentation masks for pancreas and tumor captured at Memorial Sloan Kettering Cancer Center. Seventy percent (282) of these images have been released publicly as the training set for the 2018 MICCAI Medical Decathlon Challenge^4^ (Simpson et al., 2019). This dataset is also class unbalanced, the background being the most prominent class, followed by the organ (pancreas) and the tumor as the least present class. Appearance is quite heterogeneous for pancreas and tumor. As before, the inclusion of the dice term in the loss function helps to mitigate the negative effects of such unbalance.

We perform five-fold cross-validation during training: 26 images are used for validation and 105 images for training. The mean patient shape after the resampling is 96 × 512 × 512 pixels in the case of high-resolution and 88 × 299 × 299 pixels in low-resolution. According to the median shapes, we do 3, 5, and 5 downsampling for each respective image axis in high-resolution and 3, 5, 5 downsamplings in low-resolution. The patch size in case of high-resolution is 40×192×160 pixels and 64×128×128 pixels for low-resolution.

### 3.2. Visualization of the Activation Maps

The network design allows us to visualize meaningful activations maps from the attention gates as well as from the deep supervision layers. The visualizations enable an exciting insight into the functionality of the convolutional network. The understanding of how the model represents the input image at the intermediate layers can help to gain more insight into improving the model and uncover at least part of the black-box behavior for which the neural networks are also known.

#### 3.2.1. Visualization of the Attentional Maps

**Figure 3:**
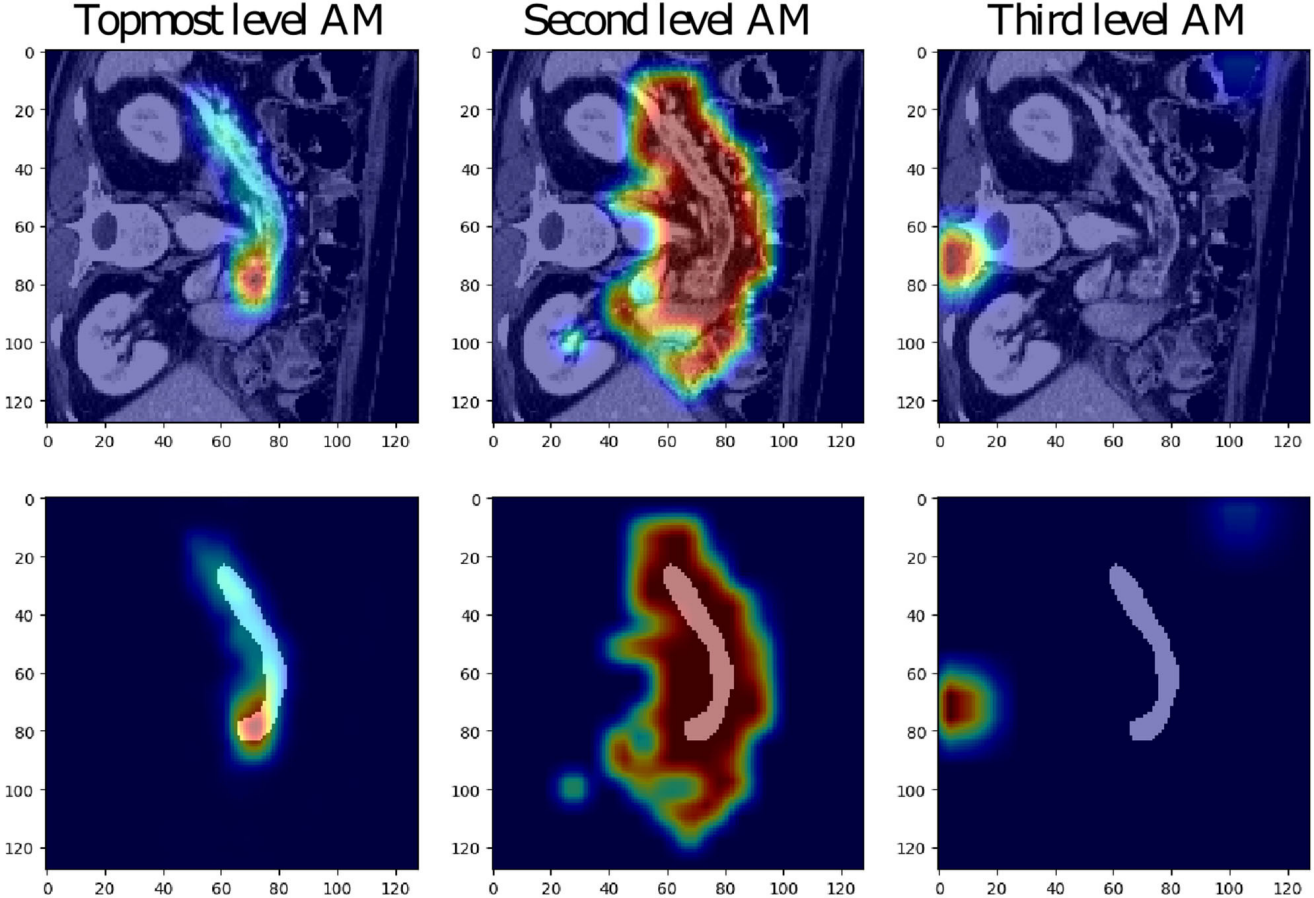
Examples of attention maps (AM) obtained from attention gates in the three topmost levels of the low-resolution VNet (from left to right: full spatial resolution, downsampling of two and four).

The low-resolution VNet was chosen to study the attention coefficients generated at different levels of a network trained on the Medical Decathlon Pancreas dataset. Figure 3 shows the attention coefficients obtained from three top network levels (working with full spatial resolution and downsampled two and four times). The attention gates provide a rough outline of the organs in top two network levels, but not in the lower spatial resolution cases. For this reason, in our experiments, we decided to implement the AG only at two topmost levels and save the computation memory to handle larger image patches.

**Figure 4:**
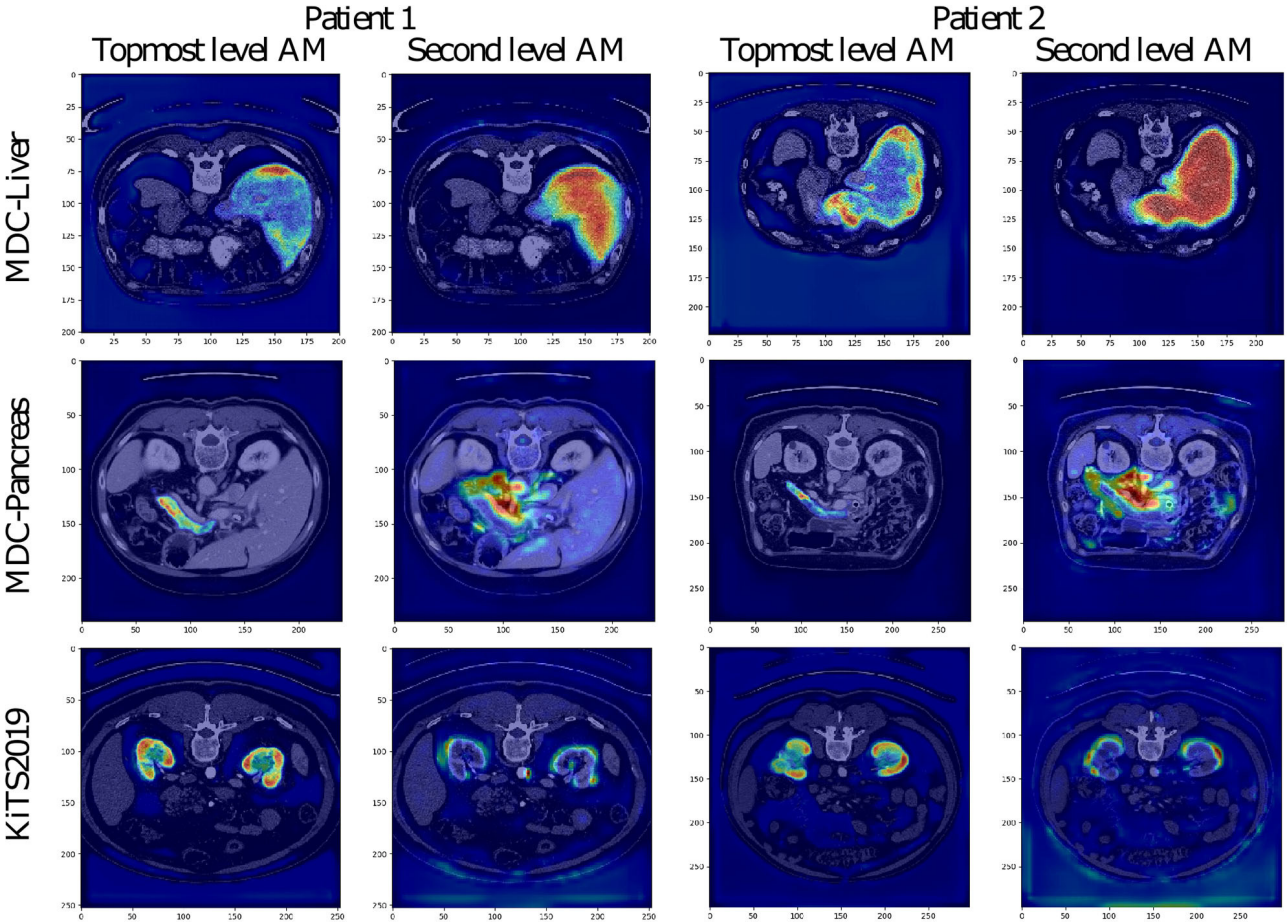
Visualization of attention maps (AM) in low-resolution for VNet and two randomly chosen patient images from the validation set of each studied dataset. For each patient, the left picture shows the attention from the topmost layer (with the highest spatial resolution), and the right picture shows the attention from the second topmost layer.

The attention coefficients obtained from two randomly chosen validation images from each studied dataset are visualized in Figure 4. All visualized attention maps correlate with the organ of interest, which indicates that the attention mechanism is focusing on the areas of interest, i.e., it emphasizes the salient image regions and significant features relevant for organ segmentation. In the case of liver segmentation, the attention map correlates accurately with the organ on the second level while in the top-level, the attention seems to focus on the organ borders. In kidney and pancreas datasets, we can observe exactly the opposite behavior. The attention map from top-level covers the organ, and the second level attention map focuses on the borders and the close organ surroundings. This difference is possibly associated with the different target sizes as the liver is taking a substantially larger part of the image than the kidney or pancreas.

#### 3.2.2. Visualization of the Deep Supervision Segmentation Maps

**Figure 5:**
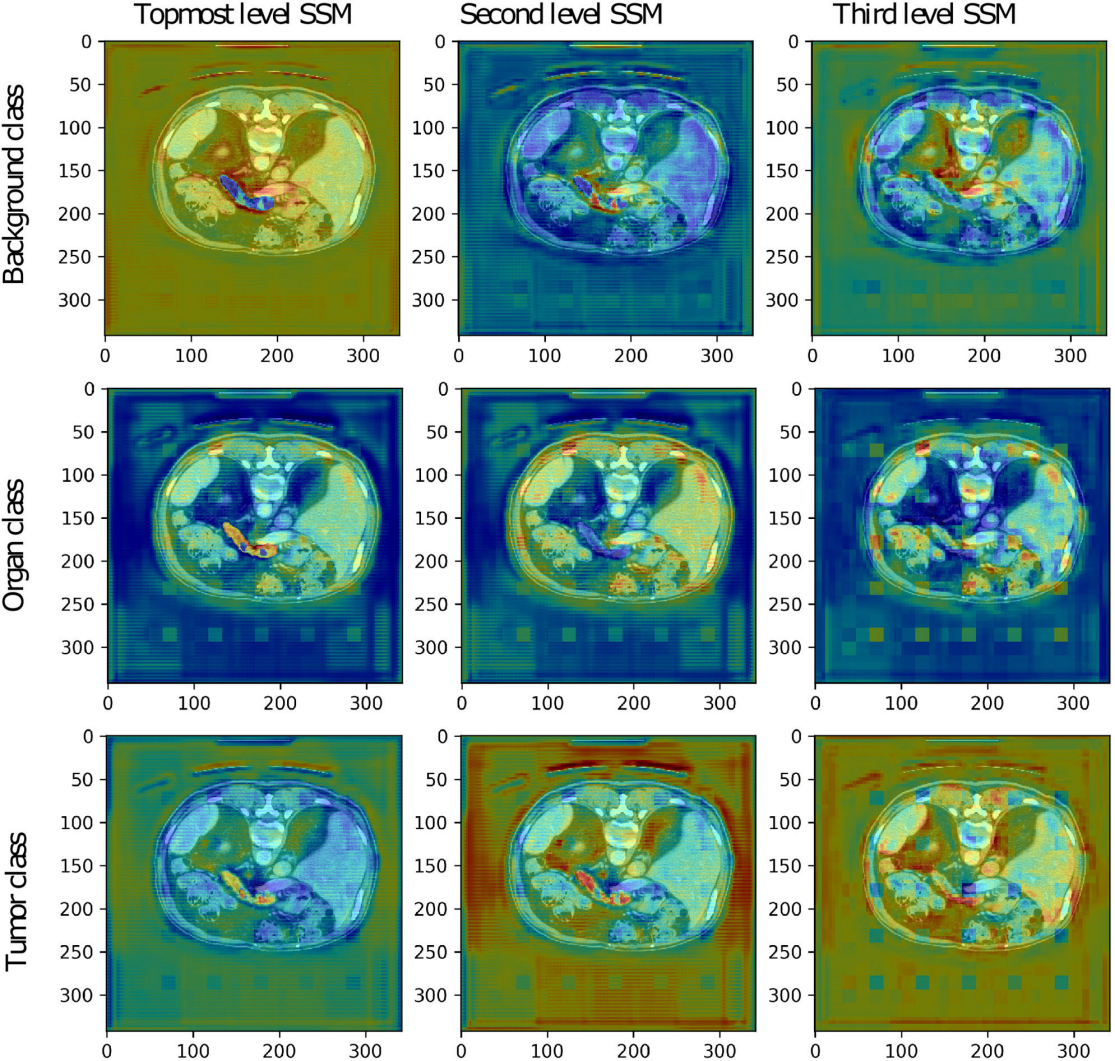
The secondary segmentation maps (SSM) obtained from deep supervision layers of low-resolution VNet for one randomly chosen patient image from the validation set of the Medical Decathlon Pancreas dataset.

The low-resolution VNet was also chosen to study the secondary segmentation maps created at lower levels of the network trained on the Medical Decathlon Pancreas dataset. The segmentation maps are shown in Figure 5. Although the primary aim of the secondary segmentation maps is not the refinement of the final segmentation created at the last layer of the model, we could see the correlation between the occurrence of each label and the activation in the segmentation maps. The topmost segmentation map copies the final output. The second and third levels of activation are noisier, as it would be expected. We could see higher activations around the pancreas in the tumor class channels and also higher activations around the borders of the organ in the background label channel.

The more in-depth segmentation maps in the organ label channel are more challenging to interpret. The second level map seems to be inverted, including the pancreas into a darker part of the input image. On the other hand, the third level map highlights all the organs present in the image. After a summation of these two maps, we achieve the desired highlight of the pancreas. Overall, we could say that all the secondary segmentation maps have a relevant impact on the final result.

### 3.3. Evaluation Metrics

We use the following metrics score to evaluate the final segmentation in the subsequent sections: precision, recall, and dice. Each of the metrics is briefly explained below.

In the context of segmentation, precision, and recall compare the results of the classifier under test with the ground-true segmentation by a combination the true positives (*T*_*P*_), true negatives (*T*_*N*_), false positives (*F*_*P*_), and false negatives (*F*_*N*_). The terms positive and negative refer to the classifier's prediction, and the terms true and false refer to whether that prediction corresponds to the ground-truth labels. To summarize, Precision *P* (7) and Recall *R* (8) are determined as follows:

(7) $$\begin{array}{l} P=\frac{T_{P}}{T_{P}+F_{P}}*100, \end{array}$$

(8) $$\begin{array}{l} R=\frac{T_{P}}{T_{P}+F_{N}}*100. \end{array}$$

This way both the precision and recall are normalized in the range 〈0, 100〉, higher values indicating better performance.

When applied to a binary segmentation task, the dice score evaluates the degree of overlap between the predicted segmentation mask and the reference segmentation mask. Given binary masks, U and V, the Dice score *D* (9) is defined as:

(9) $$\begin{array}{l} D=\frac{2*|U\cup V|}{\left|U|+|V\right|}*100. \end{array}$$

In this variant, the dice score lays in the range 〈0, 100〉, higher values indicating better performance.

### 3.4. Evaluating Four Architectures and Three Datasets

Next, we present a comprehensive study of the organ and tumor segmentation tasks on the three different abdominal CT datasets. For each dataset, four model variants were trained to show the impact of the different model architecture choices. The UNet utilizes max-pooling and the upsampling layers, while VNet is fully convolutional. Each architecture variant was trained on two different image resolutions: full-resolution and low-resolution. For more details about the model variants, please refer to section 2.2. Moreover, we provide assembly results from the respective full and low-resolution models. The soft-max output maps from the full and the low-resolution model variant were averaged and only then the final segmentation map was created. Tables 2–4 summarize the results from five-fold cross-validation for all model variants for the Medical Decathlon Challenge (MDC) Liver dataset, the Medical Decathlon Challenge Pancreas dataset and the Kidney Tumor Segmentation Challenge (KiTS) dataset, respectively.

**Table 2:** Kidney Tumor Challenge 2019.

**Architecture**		**Kidney label**			**Tumor label**		
		**Precision**	**Recall**	**Dice**	**Precision**	**Recall**	**Dice**
UNet	Low Res.	94.96 ± 0.02	96.22 ± 0.08	95.50 ± 0.01	81.51 ± 2.30	82.62 ± 3.85	79.27 ± 0.30
	Full res.	95.55 ± 0.75	97.08 ± 1.21	96.21 ± 0.62	78.83 ± 5.21	81.44 ± 4.63	76.70 ± 2.46
	Assembly	96.22 ± 1.32	97.11 ± 1.87	96.25 ± 1.12	83.88 ± 3.01	81.50 ± 6.23	78.68 ± 5.93
VNet	Low res.	94.79 ± 0.78	95.07 ± 1.42	94.63 ± 0.88	77.85 ± 3.43	78.51 ± 2.79	74.12 ± 2.66
	Full res.	96.01 ± 0.71	96.15 ± 1.19	95.93 ± 0.54	78.77 ± 3.60	79.72 ± 2.57	75.43 ± 1.59
	Assembly	96.54 ± 1.06	96.63 ± 1.35	96.43 ± 1.06	82.71 ± 2.80	83.39 ± 8.21	79.94 ± 5.33

*Metrics scores from five-fold cross validation*.

**Table 3:** Medical Decathlon Challenge 2018—Task03-Liver.

**Architecture**		**Liver label**			**Tumor label**		
		**Precision**	**Recall**	**Dice**	**Precision**	**Recall**	**Dice**
UNet	Low res.	95.01 ± 0.92	95.52 ± 1.38	94.91 ± 1.57	63.65 ± 4.92	58.13 ± 7.66	53.27 ± 4.57
	Full res.	95.39 ± 1.03	96.28 ± 1.09	95.80 ± 1.16	58.24 ± 7.23	76.39 ± 9.51	58.87 ± 3.01
	Assembly	95.95 ± 0.70	96.66 ± 1.68	96.28 ± 1.01	63.74 ± 9.51	72.86 ± 10.1	60.29 ± 3.85
VNet	Low res.	94.96 ± 0.87	95.19 ± 1.75	94.54 ± 1.97	65.17 ± 5.69	59.13 ± 11.5	54.72 ± 6.11
	Full res.	94.39 ± 1.23	95.59 ± 1.03	94.86 ± 1.25	61.12 ± 8.33	70.34 ± 9.36	57.74 ± 2.20
	Assembly	95.57 ± 0.65	95.80 ± 1.36	95.74 ± 0.89	73.42 ± 5.76	67.41 ± 13.0	64.70 ± 3.08

*Metrics scores from five-fold cross validation*.

**Table 4:** Medical Decathlon Challenge 2018—Task07-Pancreas.

**Architecture**		**Pancreas label**			**Tumor label**		
		**Precision**	**Recall**	**Dice**	**Precision**	**Recall**	**Dice**
UNet	Low res.	80.39 ± 1.83	83.70 ± 2.02	80.96 ± 2.33	62.18 ± 3.35	58.12 ± 6.12	54.66 ± 4.54
	Full res.	80.88 ± 1.66	83.77 ± 0.59	81.15 ± 0.43	60.86 ± 1.41	54.36 ± 3.76	51.66 ± 4.70
	Assembly	81.21 ± 0.62	84.51 ± 1.87	81.81 ± 0.98	62.98 ± 3.74	55.84 ± 1.42	52.68 ± 1.89
VNet	Low res.	79.36 ± 2.14	82.24 ± 1.71	79.62 ± 1.22	60.53 ± 2.72	55.19 ± 2.85	52.56 ± 2.89
	Full res.	79.92 ± 1.05	82.73 ± 1.37	80.09 ± 0.95	64.46 ± 5.23	51.30 ± 3.56	50.14 ± 4.14
	Assembly	80.61 ± 0.37	84.10 ± 1.45	81.22 ± 0.64	64.62 ± 3.29	54.39 ± 1.26	52.99 ± 2.05

*Metrics scores from five-fold cross validation*.

Due to the prominent inter-variability of position, size, and morphology structure, the tumor labels segmentation was less successful than the organ segmentation. We can see lower score values and also more significant inter-variability between the folds. The variability is especially high in the Liver-tumor label, where the lesions are usually divided into many small occurrences, and missing some of them means a significant change in the segmentation score results. The model could benefit from some postprocessing, which may help to sort out some of the lesions outside the liver organ, as suggested in Bilic et al. (2019). The overall scores are the lowest for the MDC Pancreas dataset. The variability in shape and size of the pancreas makes its segmentation a challenging task. Nevertheless, the attention mechanism helps the network to find the pancreas, thus obtaining a reasonably good performance.

Generally, the performance of the UNet and the fully convolutional VNet is comparable, but we could observe slightly better scores achieved by VNet in the MDC Liver dataset and KiTS dataset while the trend is opposed in the MDC Pancreas dataset, where the UNet provided better results than the VNet. Still, when it comes to the assembly results, the VNet benefits from its trainable parameters and achieves better results than UNet variant in all three datasets.

### 3.5. Performance Comparison

The proposed network architecture was benchmarked against the winning submission of the Medical Decathlon Challenge (MDC), namely nnUNet (Isensee et al., 2018) on two tasks: Task03-Liver and Task07-Pancreas. Table 5 shows the mean dice scores from five-fold cross-validation for the low and the full-resolution variants of models as well as the best model presented in either work. The winning results from nnUNet consist of the combined prediction from three different models (2D UNet, 3D UNet, and 3D UNet cascade) assembled together. Therefore, we have chosen to compare also the results from 3D UNet model, whose model architecture is close to our network to highlight the difference gained by the network architecture changes, namely attention gates and deep supervision.

**Table 5:** Comparison of the proposed VNet-AG-DSV to the state-of-the-art network with similar parameters presented by Isensee et al. (2018).

	**MDC task03-liver**		**MDC task07-pancreas**	
**Model**	**Liver**	**Tumor**	**Liver**	**Tumor**
	**label**	**label**	**label**	**label**
Isensee et al. (2018)—Low res.	**94.69**	47.01	79.45	49.65
Isensee et al. (2018)—Full res.	94.11	**61.74**	77.69	42.69
Isensee et al. (2018)—Best model	95.43	61.82	79.30	52.12
VNet-AG-DSV—Low res.	94.54	**54.72**	**79.58**	**52.43**
VNet-AG-DSV—Full res.	**95.95**	57.65	**80.09**	**50.14**
VNet-AG-DSV—Best model	**95.74**	**64.70**	**81.22**	**52.99**

*All the models were trained on the same dataset, released by Medical Decathlon Challenge (MDC) and validated in five-fold cross-validation. Higher score from the comparison of the two models is highlighted in bold*.

The full- and the low-resolution models with attention gates (VNet-AG-DSV) achieved higher dice scores for both labels on the pancreas dataset, of particular interest is that the tumor dice scores were substantially increased, by three and seven points in low and full-resolution, respectively. In the case of the liver dataset, we could see a significant improvement in the low-resolution case. Attention gates improved the tumor dice score by seven points while the liver segmentation precision was comparable. The decrease in dice score happened only on the tumor class in the full-resolution case. Finally, if we compare the best models presented in both papers, our model with attention gates and deep supervision (VNet-AG-DSV) wins on both datasets, adding nearly three score points on the liver-tumor class and two points in pancreas label.

The performance of the model with and without the attention gates is quantitatively compared in Table 6. We could see that both the number of parameters and the training and evaluation time increased just slightly, while the performance improvement was considerable. We should mention that the decrease in the number of parameters in the work of Isensee et al. (2018) was compensated by training the network with larger patch size: 128×128×128 pixels versus 96×128×128 pixels for the Liver dataset and 96 × 160 × 128 pixels versus 64 × 128 × 128 pixels for the Pancreas dataset.

**Table 6:** Performance comparison.

	**UNet**	**UNet-AG-DSV**	**VNet**	**VNet-AG-DSV**
Num. parameters [M]	26.2453	26.2917	29.6873	29.7383
Train iteration^*^ [ms]	224.8231	260.6527	297.2699	338.3336
Eval iteration^*^ [ms]	189.7215	217.5776	268.6558	299.3836

* *Measured as mean from 100 runs on GeForce GTX 1080 Ti*.

### 3.6. Comparison to the State-of-the-Art

The proposed architecture was evaluated on three publicly available datasets: Task03-Liver, Task07-Pancreas from Medical Decathlon Challenge and the Kidney Tumor Segmentation 2019 Challenge dataset to compare its performance with state-of-the-art methods. Next three subsections summarize the results for each dataset.

#### 3.6.1. Kidney

Our VNet with attention gates and deep supervision (VNet-AG-DSV) for the kidney-tumor task (Table 7) participated in the Kidney Tumor Segmentation Challenge of 2019, achieving a dice score 96.63 and 79.29 for kidney and tumor label, respectively, similar to our five-fold cross-validation values of 96.43 ± 1.06 and 79.94 ± 5.33 for kidney and renal tumor, respectively. The results show the stable transfer of values from validation to test set, which supports the stability of the model results. Table 7 shows the test set results for three wining submissions compared to our model. The winning solution by Isensee and Maier-Hein (2019) uses residual 3DUNet. The major difference from our solution (apart from architectural model changes) is in the loss function, which was accommodated to fit the challenge scoring system. The authors also excluded some cases from the training set (this was allowed by organizers). Second (Hou et al., 2019) and third (Mu et al., 2019) submission in KiTS challenge use some variant of a multi-step solution, where the approximate position of the kidneys is determined in the first step and only then is produced the final precise segmentation map. Please note that we performed nor manual tweaking of the training set nor any accommodation to the challenge. We can then conclude that our VNet-AG-DSV showed remarkable performance with the same architecture that was used for the other two previous tasks, namely detecting two other organs (pancreas and liver) along with their tumors (of a different structure to the kidney).

**Table 7:** Test set results from the Kidney Tumor Challenge 2019 leaderboard.

**Team**	**Composite dice**	**Kidney dice**	**Tumor dice**
Isensee and Maier-Hein (2019)	91.23	97.37	85.09
Hou et al. (2019)	90.64	96.74	84.54
Mu et al. (2019)	90.25	97.29	83.21
VNet-AG-DSV	87.96	96.63	79.29

#### 3.6.2. Liver

The liver-tumor dataset was obtained from the Medical Decathlon Challenge (MDC) happening at the MICCAI conference in 2018. We analyze the results from various research papers dealing with liver and liver-tumor segmentation. The Bilic et al. (2019) in work Liver Tumor Segmentation Benchmark (LiTS) presents a comparative study of two challenges dealing with liver and liver-tumor segmentation. Authors note that not a single algorithm performed best for liver and tumors simultaneously. The winner of liver segmentation, Tian et al. achieves the dice score 96.30 and 65.70 for liver and tumor class, respectively. The winner of the lesion segmentation part, Yuan et al. gained the dice score of 96.10 and 70.20 for the liver and tumor classes, respectively. All winning methods in LiTS benchmark utilized some post-processing steps, most commonly the connected component labeling but also other methods more specific for the concrete task of liver lesion detection. As shown in Table 8, our VNet-AG-DSV achieved the dice scores 96.37 and 64.70 for liver and tumor class, respectively. Our method, being fully automatic and not using hand-tuned post-processing, not only provides comparable results, it can also be easily transferred and used on different organ segmentations task as shown next.

**Table 8:** Comprarison of the state-of-the-art methods for liver and liver-tumor segmentation from CT scans.

**Team**	**Composite Dice**	**Liver Dice**	**Tumor Dice**
Bilic et al. (2019)	83.15	96.10	70.20
Bilic et al. (2019)	81.00	96.30	65.70
Isensee et al. (2018)	78.63	95.43	61.82
VNet-AG-DSV	80.56	96.37	64.70

* *The models were trained and tested on different dataset*.

#### 3.6.3. Pancreas

In comparison to other abdominal organs, the pancreas segmentation is a challenging task, as shown by the lower dice scores achieved in the literature. Roth et al. (2018) introduces an application of holistically-nested convolutional networks (HNNs) and achieves the dice score 81.27 ± 6.27. Oktay et al. (2018) introduces the attention gates for pancreas segmentation but compared to our solution does not include deep supervision while differing in other architectural choices. Their network achieves the dice score 84.00 ± 8.70 for the pancreas label. To best of our knowledge, there exist no papers dealing with both, pancreas and pancreas-tumor segmentation, except the ones submitted for the Medical Decathlon Challenge. The best dice score for the pancreas, and the pancreas-tumor segmentation, achieved in this challenge by Isensee et al. (2018) is 79.30 and 52.12, respectively. As shown in Table 9, the dice scores from our VNet-AG-DSV are 81.22 and 52.99 for pancreas and tumor label, respectively. Our method beats the nnUNet by Isensee et al. (2018) in both labels, and its pancreas segmentation result equals to the methods dedicated only to pancreas detection.

**Table 9:** Comprarison of the state-of-the-art methods for pancreas and pancreas-tumor segmentation from CT scans.

**Team**	**Composite Dice**	**Liver Dice**	**Tumor Dice**
Roth et al. (2018)^*^	-	81.27	-
Oktay et al. (2018)^*^	-	84.00	-
Isensee et al. (2018)	65.71	79.30	52.12
VNet-AG-DSV	67.11	81.22	52.99

* *The models were trained and tested on different dataset*.

## 4. Discussion

Conventional artificial neural networks with fully connected hidden layers take a very long time to be trained. Due to this, the convolutional neural network (CNN) was introduced. It is specifically designed to work with the images by the use of convolutional layers and pooling layers before ending with fully connected layers. Nowadays, convolutional neural network architectures are the primary choice for most of the computer vision tasks. CNN takes inspiration in biological processes in that the connectivity pattern between neurons corresponds to the organization of the animal visual cortex (Hubel and Wiesel, 1968; Fukushima, 1980; Rodŕıguez-Sánchez et al., 2015). Similarly, as in the eye, individual neurons respond to stimuli from a restricted (bounded by the filter size) region of the visual field. These restricted receptive fields of different neurons partially overlap, and together they cover the entire visual field.

Image segmentation is one of the most laborious tasks in computer vision since it requires the pixel-wise classification of the input image. Long et al. (2015) presents a cully convolutional neural network for image segmentation, firstly introducing the skips between layers to fuse coarse, semantic and local, appearance information. The work of Ronneberger et al. (2015) extended the idea of skip connections and applied it favorably in medical image segmentation. The possibility to examine the image at different image scales proved to be crucial in successful image segmentation. Due to a volume characteristic of medical data, the 3D variant of fully convolutional networks with skip connections was introduced by Milletari et al. (2016). This type of architecture is the most used CNN in the field of medical image segmentation since then, scoring best at most leading challenges dealing with the medical image segmentation in the last years: The Liver Tumor Segmentation Challenge in 2017 (Bilic et al., 2019), the Medical Decathlon Challenge in 2018 (Simpson et al., 2019), and the Kidney Tumor Segmentation Challenge in 2019 (Heller et al., 2019).

The deep supervision presented by Kayalibay et al. (2017) takes the idea of skip connections and uses it differently. It is a design where multiple segmentation maps are generated at different resolutions levels of the network. The feature maps from each network level are transposed by 1 × 1 × 1 convolutions to create secondary segmentation maps. These secondary maps are not intended for any further refinement of the final segmentation map. Instead, it tries to correct the earlier layers of the network and “encourage” them to produce better segmentation results, thus speeding the convergence at training. The deep supervision is especially useful in tackling the problem of the vanishing gradient, which usually occurs during the training of very deep CNN.

Apart from the skip connections, many researches tried to incorporate the concept of attention into artificial CNN visual models (Mnih et al., 2014; Xiao et al., 2015; Xu et al., 2015; Chen et al., 2016). The presence of attention is one of the unique aspects of the human visual system (Corbetta and Shulman, 2002), which helps to selectively process the most relevant part of the incoming information for the task at hand. (Chen et al., 2016) proposes an attention model that softly weights the features from different input scales when predicting the semantic label of a pixel. Oktay et al. (2018) utilized a similar principle in their attention gates and applied them in medical image segmentation. Attention is especially helpful in the case of internal organ segmentation from abdominal computed tomography (CT) scans because abdominal organs are characteristically represented by similar intensity voxels in CT scans. The model greatly benefits from the ability to discard the activation from insignificant parts of the image and focus on the organ of interest. Eventually, the human expert would follow the same methodology: first, find the rough position of the organ of interest and only then analyze it in detail, as could be found in the description of the segmentation maps annotating process for the KiTS challenge (Heller et al., 2019).

## 5. Conclusions

This work presents a comprehensive study of medical image segmentation via a deep convolutional neural network. We propose a novel network architecture extended by attention gates and deep supervision (VNet-AG-DSV) which achieves results comparable to the state-of-the-art performance on several and very different medical image datasets. We performed extensive study which analyze the two most popular convolutional neural networks in medical images (UNet and VNet) across three different organ-tumor datasets and two training image resolutions. Further, to understand how the model represents the input image at the intermediate layers, the activation maps from attention gates and secondary segmentation maps from deep supervision layers are visualized. The visualizations show an excellent correlation between the activation present and the label of interest. The performance comparison shows that the proposed network extension introduces a slight computation burden, which is outweighed by considerable improvement in performance. Finally, our architecture is fully automatic and has shown its validity at detecting three different organs and tumors, i.e., more general than the state of the art, while providing similar performance to more dedicated methods.

## Data Availability Statement

Publicly available datasets were analyzed in this study. This data can be found here: http://medicaldecathlon.com/, https://kits19.grand-challenge.org/.

## Author Contributions

AT and TT coded the proposed methodology and performed the experiments. ZK helped to ensure the needed computation power. AT wrote the first draft of the manuscript. AR-S did the first approval reading. All authors contributed conception and design of the study, contributed to manuscript revision, read, and approved the submitted version.

## Conflict of Interest

The authors declare that the research was conducted in the absence of any commercial or financial relationships that could be construed as a potential conflict of interest. The handling Editor declared a past co-authorship with one of the authors AR-S.

## Footnotes

^1^For example website Grand Challenges in Biomedical Image Analysis gathers multiple competitions; https://grand-challenge.org.

^2^A one-hot encoding was created from the original ground true segmentation map in a way, that each image channel contains only one class present in segmentation map, this way all the classes are represented by value one just in different image channels. For example, if we have ground true segmentation map of size (1 × *imSize*1 × *imSize*2 × *imSize*3) with three labels: 0, 1, 2. The one-hot encoding would have the size (3 × *imSize*1 × *imSize*2 × *imSize*3).

^3^kits19.grand-challenge.org

^4^medicaldecathlon.com
